# Direct Detection and Identification of the Most Common Bacteria and Fungi Causing Otitis Externa by a Stepwise Multiplex PCR

**DOI:** 10.3389/fcimb.2021.644060

**Published:** 2021-03-25

**Authors:** Shima Aboutalebian, Kazem Ahmadikia, Hamed Fakhim, Javaher Chabavizadeh, Ahmadreza Okhovat, Mahnaz Nikaeen, Hossein Mirhendi

**Affiliations:** ^1^ Department of Medical Parasitology and Mycology, School of Medicine, Isfahan University of Medical Sciences, Isfahan, Iran; ^2^ Department of Medical Parasitology and Mycology, School of Public Health, Tehran University of Medical Sciences, Tehran, Iran; ^3^ Infectious Diseases and Tropical Medicine Research Center, Isfahan University of Medical Sciences, Isfahan, Iran; ^4^ Department of Otolaryngology, Isfahan University of Medical Sciences, Isfahan, Iran; ^5^ Department of Environmental Health Engineering, School of Health, Isfahan University of Medical Sciences, Isfahan, Iran; ^6^ Core Facilities Laboratory (CFL), Mycology Reference Laboratory, Isfahan University of Medical Sciences, Isfahan, Iran

**Keywords:** Otitis externa, detection and identification, multiplex PCR, fungi, bacteria

## Abstract

**Background:**

Considering the importance of differential diagnosis of infectious otitis externa (OE), a stepwise PCR-based assay using universal and genus- or species-specific primers for the detection/identification of the most prevalent bacterial and fungal OE was developed and evaluated on the ear aspiration specimens of clinically suspected patients.

**Methods and Materials:**

A total of 120 ear aspiration specimens with otomycosis suspicion were subjected to manual DNA extraction using phenol–chloroform extraction after tissue digestion with a lysis buffer. The multiplex PCR was initially performed using pan-fungal and bacterial homemade primers. *Pseudomonas* and *Staphylococcus* specific primers were simultaneously used in one reaction mixture to identify the bacterial genera. Furthermore, for the identification of fungal agents, *Candida* species-specific multiplex primers targeting the most clinically important *Candida* species causing OE (*i.e*., *C. albicans*, *C. parapsilosis*, and *C. auris*), as well as *Aspergillus* related multiplex PCR identifying the most prevalent *Aspergillus* species were used in two separate reaction mixtures. All the results of multiplex PCR were interpreted based on the amplicon size.

**Results:**

The overall multiplex PCR-based detection rate of bacterial (n = 88; 73.3%) and fungal (n = 97; 81%) OE was documented to be 100% along with and complete consistency with the results of direct examination and Giemsa staining. Double amplicon bands of bacterial and fungal pathogens were evidenced in 76 specimens (63.3%). Moreover, the positivity rate of pan-fungal PCR was higher than that of the culture result. Out of 88 pan-bacterial positive PCR specimens, 66 and 47 ones were positive for *Staphylococcus* and *Pseudomonas*, respectively. In addition, 30 samples exhibited mixed infection of both, and five specimens remained negative. Out of 97 pan-fungal positive PCR specimens, 67 and 51 ones contained *Candida* and *Aspergillus* species, respectively. It should be noted that dual amplicon bands of *Candida* and *Aspergillus*-related multiplex PCR were yielded in 30 specimens.

**Conclusion:**

The stepwise multiplex PCR assay proved to be more sensitive, more rapid, as well as less cumbersome in detection and identification of fungal and bacterial OE, compared to culture.

## Introduction

Otitis externa (OE) describes a group of acute or chronic inflammatory disorders of the external auditory canal and auricle following disruption of the protective squamous epithelial layer of the ear canal (Thorne and Wetmore, 2009). The inflammation may have arisen from an allergic (reactive) or infective (bacteria, fungi, and viruses) origin (Salyer, 2007; Adegbiji et al., 2017). Considering an annual incidence rate of 1.2% (Rowlands et al., 2001), OE is a common infectious condition for which patients attend Ear, Nose, and Throat clinics to undergo medication therapy. Different types of risk factors can predispose individuals to develop OE, including water trapping in the ear canal due to swimming, humidity, or sweating; cerumen, and epithelial disintegration as a result of increase in pH; cerumen removal; hearing aids and instrumentation or itching; canal obstruction because of foreign body or sebaceous cyst; anatomic abnormalities; dermatologic conditions, such as eczema, psoriasis, or seborrhea; prolonged antibiotic usage; and diabetes status (Schaefer and Baugh, 2012). Pseudomonas aeruginosa and *Staphylococcus aureus* are frequently listed as the most predominant agents causing an acute form of OE (Schaefer and Baugh, 2012). On the other hand, fungal agents, most commonly *Aspergillus* and *Candida* species, are associated with prolonged antibiotic usage and chronic OE (Sarwestani et al., 2018; Aboutalebian et al., 2019; Punia et al., 2019). Complaints of acute onset of unilateral ear pain, itching, and a sense of aural fullness are the typical manifestations of acute otitis externa caused by bacteria. However, in fungal ear infections (otomycosis), pruritus with or without associated otorrhea are frequently presented, the symptoms of which are more chronic than bacterial infections; moreover, pain is usually not a prominent sign (Thorne and Wetmore, 2009). Given the different management for diverse microbial agents causing ear infections, precise detection/identification of causative agents is a matter of great importance for accurate prognosis prediction and optimizing effective therapy (Schaefer and Baugh, 2012). Although the diagnosis of ear infection is routinely established through a clinical examination and history review (Thorne and Wetmore, 2009), the correct diagnosis is hampered in case of uncooperative patients (*i.e.*, children), unspecific clinical diagnosis, need for targeted therapy, refractory OE, co-infection, and inexperienced clinicians (Chonmaitree and Henrickson, 2000; Blomgren and Pitkäranta, 2005). Therefore, laboratory-based methods for reliable diagnosis are required in these circumstances. Direct examination and culture are still the cornerstones of bacterial and fungal diagnosis. Nevertheless, direct or histopathological examinations take a longer time to establish the diagnosis and face difficulties in accurate identification of the infecting agent and may need personal expertise. In the former study, it has been evidenced that the culture positivity rate of the agents causing fungal infections is as low as 50%, especially when *Malassezia* species or *Mucorales* are highly suspected (Roden et al., 2005). In many cases, fungi fail to grow due to pretreatment, insufficient volume of specimens, or lack of specific culture medium (Zhao et al., 2011; Zaman et al., 2017). The development of rapid molecular-based techniques, mainly polymerase chain reaction (PCR) assays, has provided and improved the possibility for the detection of small numbers of microbial genomes in fresh specimens, particularly for tissue with negative culture or slow-growing pathogens (Jalali et al., 2008; Gruber et al., 2015). Therefore, to overcome the low isolation rate of culture and difficulties in the identification of causative agents by clinical evaluation and direct examination, PCR-based methods have been recommended in different studies (Bialek et al., 2005; Rickerts et al., 2007; Hammond et al., 2011). Although sequencing of common barcoding regions has been proved to be the most reliable pathogen identification tool in developed countries that is used routinely in laboratories, its provision is not easy to afford in developing and low resources countries. Moreover, serially performing a separate PCR assay as routine use for the detection of individual pathogens is labor-intensive, time-consuming, and expensive, particularly if a various panel of potential causative agents should be tested. Although several infectious diseases have been differentially diagnosed by multiplex PCR, differential diagnosis of agents causing OE has not yet been performed *via* multiplex PCR directly from clinical specimens. Therefore, this study aimed to evaluate the usefulness of novel homemade multiplex PCR capable of simultaneously detecting and identifying the most commonly isolated species of bacteria and fungi directly in the ear aspiration specimens obtained from patients suspected of OE. Moreover, it was attempted to compare the results with those of the direct microscopic examination and tissue culture techniques.

## Materials and Methods

### Patient and Isolates

A total of 120 patients clinically suspected of OE attending the otorhinolaryngology clinic of Al-Zahra Teaching Hospital, Isfahan, Iran, whose ear aspirations or ear swab specimens documented infective fungal and/or bacterial elements on direct microscopic examination by 10% Potassium Hydroxide and Giemsa mounts, were included in this study. For the identification of the yeasts isolated on CHROM agar *Candida* medium, the internal transcribed spacer (ITS) PCR-RFLP using the restriction enzymes *Msp*I was performed as described in our previous report (Aboutalebian et al., 2019). *Aspergillus*/*Penicilliu*m species which grew on the medium Sabouraud’s dextrose agar supplemented with 50 mg L^–1^ chloramphenicol, were identified by partial *β*-tubulin gene sequencing. Unidentified yeasts or molds (other than *Aspergillus/Penicillium*) were subjected to the ITS sequencing (Aboutalebian et al., 2019). Furthermore, for the identification of bacterial agents, neither culture-based methods nor molecular procedures were applied; therefore, they were blindly investigated in the present study. Written informed consent and detailed clinical data of the patient have been presented in our former report (Aboutalebian et al., 2019). The study protocol was approved by the Research Ethics Committee of Iran National Institute for Medical Research Development (IR.NIMAD.REC.1398.063).

### Multiplex PCR Designing

In order to diagnose a panel of most common bacteria and fungi causing OE, a step-by-step multiplex PCR assay was designed for simultaneous detection and differentiation of the etiological agents. The DNA sequences of the candidate genetic target for designing the primers were retrieved from the NCBI database (https://www.ncbi.nlm.nih.gov/nucleotide/). *In silico* analyses and the criteria for the selection of primers were reasoned as follows: 1) no cross-reactivity with the other species, other loci, or other primers and similar annealing features of the compatible primers, 2) compatibility of amplicon sizes and sequence complexity of one target species with the rest of target species in the same multiplex PCR, 3) compatibility of melting temperature of primers within the same multiplex PCR, and 4) primer annealing avoidance with nonspecific sequences and other species by locating the gaps and mismatches in the 3′ end of primers. Potential interfering sequences and species were assigned as a negative control for each primer pair. The 18SrDNA, internal transcribed spacer 1 (ITS1) or ITS2 regions, and *β*-tubulin gene were the subject loci for universal fungal detection, *Candida* species identification, and *Aspergillus* species identification, respectively. Additionally, the 16SrDNA region was used for designing the primers for the pan bacterial screening of bacterial agents causing OE and also for specific discrimination between *Pseudomonas* and *Staphylococcus*. Online free Primer blast and Geneious software (https://www.geneious.com) were used for primer designing. **Table 1** tabulates all primers, target loci, and estimated amplicon size of the primers. The specific primers for Staphylococcus spp. was choose according to Karimi et al. (2020).

**Table 1:** Species, target genes, and associated primers employed in the stepwise multiplex PCR.

Stepwise Multiplex		Primer	Sequence (5**′** 3**′**)	Target	Size (bp)	Positive controls	Negative controls
Step 1		U fungi	F: ACATCCAAGGAAGGCAGCAGG R: GAGTTTCCCCGTGTTGAGTCAA	18s	783–800	*A. flavus* (Clinical sample 1), *A. oryzae* (Clinical sample 88), *A. niger* (Clinical sample 107), *A. terreus *(Clinical sample 13), *A. tubingensis* (Clinical sample 18), *T. rubrum *(CBS100237), *T. schoenleinii* (*CBS434.63*), *C. tropicalis* (CBS 94), *C. albicans* (ATCC64553), *C. glabrata*(ATCC90030)	Blastocystis (*Clinical sample*), *Toxoplasma gondii* (*Clinical sample*), *Leishmania major* (*Clinical sample*), *Leishmania tropica* (*Clinical sample*), Bacillus subtilis, *Enterobacteriaceae*, *Pseudomonas aeruginosa*, *escherichia coli*, *Salmonella*, *Staphylococcus aureus, human DNA*
		U bacteria	F: ATGTTGGGTTAAGTCCCG R: CTAGCGATTCCRRCTTCA	16s	265–267	Bacillus subtilis *Enterobacteriaceae* *Pseudomonas aeruginosa* *escherichia coli* *Salmonella* *Staphylococcus aureus* *Klebsiella*	*A, flavus* (Clinical sample 9), *A. niger *(Clinical sample 107), *T. rubrum*(CBS100237), *C. albicans* (ATCC64553), *human DNA*
Step 2-1 Bacterial		U *Staphylococcus*	F: AACTCTGTTATTAGGGAAGAACA R: CCACCTTCCTCCGGTTTGTCACC	16s	756	*Staphylococcus aureus* (Clinical sample 4), *Staphylococcus aureus* (Clinical sample 8)	*A. tubingensis* (Clinical sample 18), *C. albicans* (ATCC64553)
		U *Pseudomonas*	F: TAGCCGTTGGGATCCTTGAGAT R: CCAATCCATCTCTGGAAAGT	16s	198	*Pseudomonas aeruginosa*	*A. niger *(Clinical sample 107), *C. albicans* (ATCC64553), *Toxoplasma gondii* (*Clinical sample*), *human DNA*
Step 2-2 fungal	*Candida related multiplex PCR*	alb	F: GCACCACATGTGTTTTTCTTTGAA R: TGGTGGACGTTACCGCCG	ITS	417	*C. albicans* (ATCC64553), *C. albicans* (Clinical sample 39), *C. albicans* (Clinical sample 72)	*C. tropicalis* (CBS 94), *A*. *flavus* (Clinical sample 9), *Malassezia* (Clinical sample 45), *C. glabrata*(ATCC90030), *C. auris human DNA*, *C. dubliniensis* (ATCC 2018), *C. parapsilosis* (CBS 711)
		para	F: GTA GGC CTT CTA TAT GGG GC R:GTTTATACTCCGCCTTTCTTTC	ITS	308	*C. parapsilosis* (Clinical sample 30), *C. parapsilosis* (Clinical sample 32), *C. parapsilosis* (Clinical sample 115), *C. parapsilosis* (CBS 711)	*C. tropicalis* (CBS 94), *A. flavus* (Clinical sample 9), *Malassezia* (Clinical sample 45), *C. albicans* (ATCC64553), *C. glabrata*(ATCC90030), *C. auris,human DNA*
		*auri*s	F: ATTTTGCATACACACTGATTTGG R: AACGCCACCGCGAAGATT	ITS	250	*C. auris* (ATCC *8033), C. auris* (ATCC *8037)*	*A. flavus* (Clinical sample 9), *C. tropicalis* (CBS 94), *C. albicans* (ATCC64553), *C. glabrata *(ATCC90030), *human DNA*
	*Aspergillus* related multiplex PCR	*terreus*	F: TGCAGAGTCTTACGGACATGC R: GGGATCGAAATTAGTGTCGAGGT	beta-tubulin	321	*A. terreus *(Clinical sample 13), *A. terreus* (Clinical sample 35*)*	*A. flavus* (Clinical sample 1), *A. oryzae* (Clinical sample 88), *A. niger*(Clinical sample 107), *A. tubingensis* (Clinical sample 18), *A. fumigatus*(Clinical sample 20), *C. albicans* (ATCC64553), *human DNA*
		*tubingensis*	F: GTTATCCATCGGGTATATAGC R: TCAGGGACGGTGTGATCTA	beta-tubulin	246	*A. tubingensis* (Clinical sample 16), *A. tubingensis* (Clinical sample 18), *A. tubingensis* (Clinical sample 45), *A. tubingensis* (Clinical sample 114)	*A. flavus* (Clinical sample 1), *A*. *oryzae* (Clinical sample 88), *A. niger*(Clinical sample 107), *A. terreus* (Clinical sample 13), *A. fumigatus* (Clinical sample 20), *C. albicans* (ATCC64553), *human DNA*
		*niger*	F: TCCATTAGGTACATGCTATCGG R: CCAAGGTCCGATGGATCTCA	beta-tubulin	243	*A. niger* (Clinical sample 14), *A. niger* (Clinical sample 98*), A. niger* (Clinical sample105), *A. niger* (Clinical sample 107)	*A. flavus* (Clinical sample 1), *A. oryzae * (Clinical sample 88), *A. terreus* (Clinical sample 13), *A. tubingensis* (Clinical sample 18), *A. fumigatus* (Clinical sample 20), *T. rubrum* (CBS100237), *human DNA*
		*fumigatus*	F: ATGACGGGTGATTGGGA R: CGTCCGCTTCTTCCTTGTT	beta-tubulin	199	*A. fumigatus* (Clinical sample 1), *A. fumigatus* (Clinical sample 20)	*A. flavus* (Clinical sample 1), *A. oryzae* (Clinical sample 88), *A. niger*(Clinical sample 107), *A. terreus* (Clinical sample 13), *A. tubingensis* (Clinical sample 18), *human DNA*
		*flavi*	F: TCCTCAAAAGCATGATCTCGG R:CCAACTTCTAATGCCATATGGT	beta-tubulin	166	*A. flavus* (*Clinical sample 1*), *A. flavus* (*Clinical sample 9*), *A. oryzae* (*Clinical sample 82*), *A. oryzae* (Clinical sample 88)	*A. niger* (*Clinical sample 107*), *A. terreus* (*Clinical sample 13*), *A. tubingensis* (*Clinical sample 18*), *A. fumigatus* (*Clinical sample 20*), *T. rubrum* (*CBS100237*), *C. albicans *(*ATCC64553*), *human DNA*

### DNA Extraction

The genomic DNA was manually extracted from each ear aspiration specimen as follows: About 5 mm^3^ of the clinical specimens were kept at −20°C and transferred to a 1.5-mL Eppendorf tube containing 300 mg of glass beads (0.5 mm in diameter, 400 μl of lysis buffer (100 mM Tris, pH 8; 10 mM EDTA; 100 mM NaCl; 1% sodium dodecyl sulfate; 1% Triton X-100) and 300 μl phenol–chloroform, and severely homogenized for 3 × 60 s at 6,000 rpm by a homogenizer (Bertin Instrument, Precelleys 24). After centrifugation at 5000 for 5 min, the supernatant was transferred to a new tube and 300 μl of phenol/chloroform was added, and centrifuged for 5 min at 5,000 rpm, and the supernatant was transferred to a new tube; moreover, the identical volume of the chloroform was added and centrifuged for 5 min at 5,000 rpm. In the next stage, 2.5 volumes of absolute ethanol and a 0.1-volume of 3 M sodium acetate (pH 5.2) were added to the supernatant in a new tube and incubated at −20°C for 1 h, followed by centrifugation for 10 min at 12,000 rpm. The supernatant was then discarded, and the precipitate was washed with cold ethanol (70%), dried in the air, dissolved in 30 μl of distilled water, and stored at −20°C as the purified DNA until PCR performance.

### PCR Workflow

**Figure 1:**
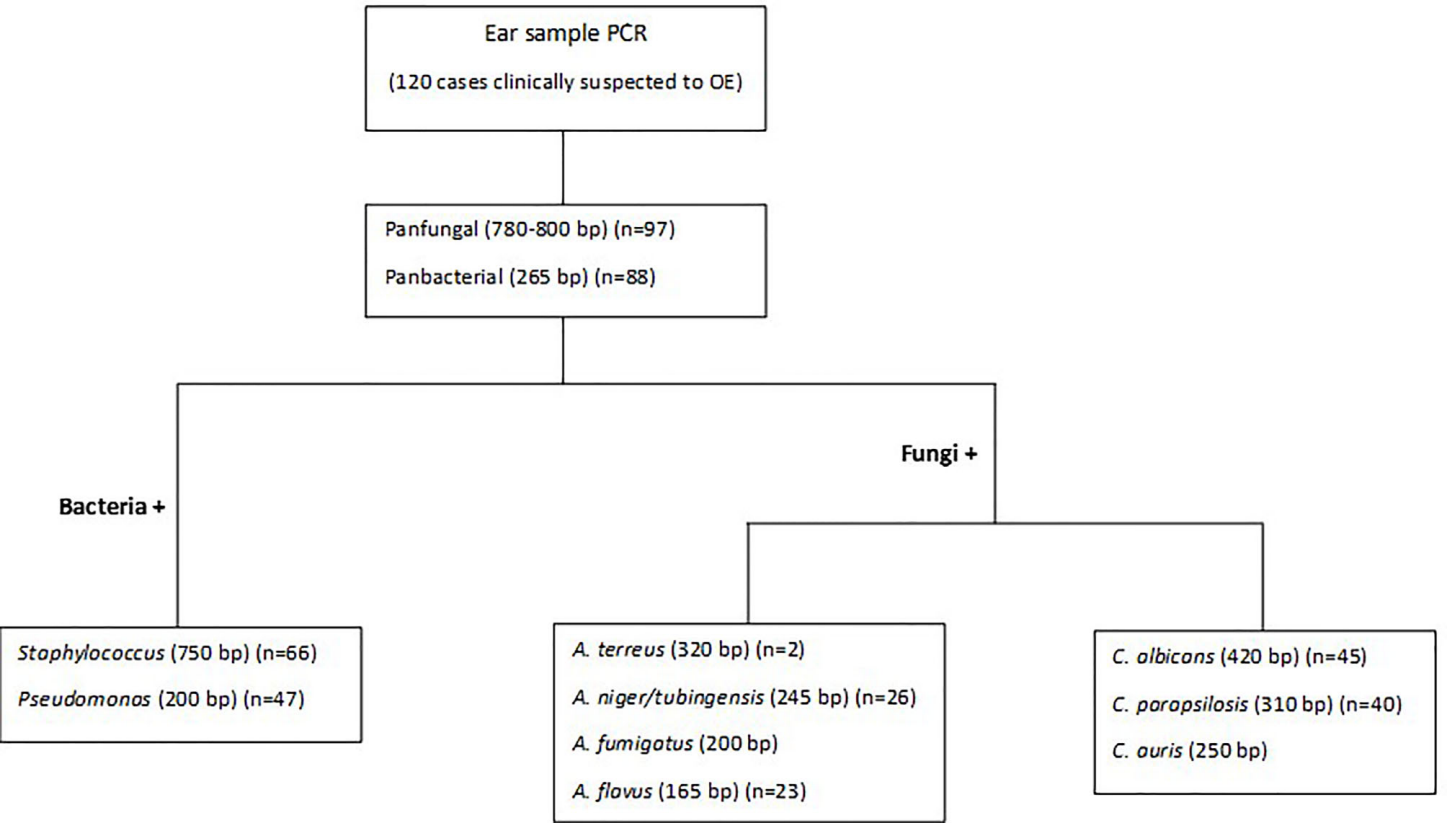
Schematic of the stepwise multiplex PCR.

Detection/Identification of bacterial and fungal agents causing OE was performed in two or three multiplex PCRs (**Figure 1**). The first PCR reaction detects and simultaneously differentiates the fungal from bacterial agents using two pan-fungal and pan-bacterial primers (duplex PCR). According to the positive result of each universal primer and amplicon size (pan-fungal: 783–800 base pair, pan-bacterial: 265–267 bp), the second PCR was selectively run (**Table 1**, **Figure 1**). Accordingly, if the first PCR was positive by pan-bacterial primer, then the second duplex PCR was applied to differentiate the most prevailing causal agents of bacterial OE, including *Pseudomonas* and *Staphylococcus* based on amplicon size (**Table 1**). On the other hand, if the first PCR was positive by the universal fungi primer (amplicon size of 783–800 bp), fungal species-specific multiplex PCRs were run, such as *Candida* multiplex PCR targeting the most clinically important *Candida* species causing OE (*i.e.*, *C. albicans*, *C. parapsilosis*, and *C. auris*), and *Aspergillus* multiplex PCR identifying the most prevalent *Aspergillus* species (*i.e.*, *A. niger, A. tubingensis, A. flavus, A. fumigatus*, and *A. terreus*) (**Tables 1** and **2**).

**Table 2:** The optimized volumes and annealing temperature for each multiplex PCR.

Multiplex PCR	Mastermix 2× (μl)	Forward primer (each primer) (μl) (concentration)	Reverse primer (each primer) (μl) (concentration)	Template DNA (μl)	water	Total reaction volume (μl)	PCR conditions			Cycle
Pan-bacterial and Pan-fungal	7.5	0.4 (0.27 μM)	0.4 (0.27 μM)	3	2.9	15	Initial Denaturation	95	5min	1
							Denaturation	94	15s	35
							Annealing	58	45s	
							Extension	72	30s	
							Final extension	72	5min	1
*Pseudomonas* and *Staphylococcus*	7.5	0.3 (0.2 μM)	0.3 (0.2 μM)	3	3.3	15	Initial Denaturation	95	5 min	1
							Denaturation	94	15 s	35
							Annealing	58	30 s	
							Extension	72	30 s	
							Final extension	72	2 min	1
*Candida* related multiplex PCR	7.5	0.4 (0.27 μM)	0.4 (0.27 μM)	2	3.1	15	Initial Denaturation	95	5 min	1
							Denaturation	94	15 s	35
							Annealing	60	30 s	
							Extension	72	30 s	
							Final extension	72	2 min	1
*Aspergillus* related multiplex PCR	10	0.4 (0.2μM) except *flavi* 0.8(0.4μM)	0.4 (0.2μM) except *flavi* 0.8(0.4μM)	5	0.2	20	Initial Denaturation	95	5 min	1
							Denaturation	94	30 s	35
							Annealing	58	45 s	
							Extension	72	45 s	
							Final extension	72	5 min	1

### Specificity Testing

For optimizing, validating, and evaluating the specificity of the multiplex PCR, some CBS and ATCC reference strains as the target species, as well as some human DNAs, strains, or DNAs of clinically important fungal, bacterial, and protozoan species as non-target, were tested and served as the panel of positive and negative (**Table 1**). The DNA extracted from healthy ears was also tested into the assay system to evaluate the possible false-positive result. The results achieved by each multiplex PCR were compared with those of species identity of each sample, direct microscopy, and culture. Eventually, the reproducibility of each obtained result was practically tested to assess the precision of each multiplex PCR. In order to monitor the presence of inhibitor or potential cross-contamination, an appropriate DNA and sterile distilled water serving as positive and negative control were employed for each PCR run.

## Results

### Specificity of the Primers

*In silico* analyses of the designed primers showed no primer mismatches with non-target organisms. Moreover, no cross-reactivity was recorded when a wide range of clinically important or environmental non-target fungal, bacterial, protozoan species, and human DNA were tested. In addition, the assay yielded the appropriate results using target DNA and sterile distilled water serving as a positive and negative control, respectively.

### PCR Assay Resolution for Species Identification

Subjecting CBS and ATCC reference strains and previously identified clinical isolates to the stepwise multiplex PCR assay led to consistent identification of each species. Additionally, as expected, cryptic species belonging to *Aspergillus* section *Nigri*, *Aspergillus* section *Flavi*, and *Aspergillus* section *Terrei* were identified as *A. niger/A. tubingensis*, *A. flavus/A. oryzae*, and *A. terreus*, respectively. However, neither cryptic species of *C. parapsilosis* complex nor rare species belonging to *Aspergillus* section *Fumigati* were tested to evaluate the designed primers identifying *C. parapsilosis* species complex and *A. fumigatus*-related species.

### PCR Versus Direct Examination for Detection/Identification of Bacterial and Fungal OE

**Figure 2:**
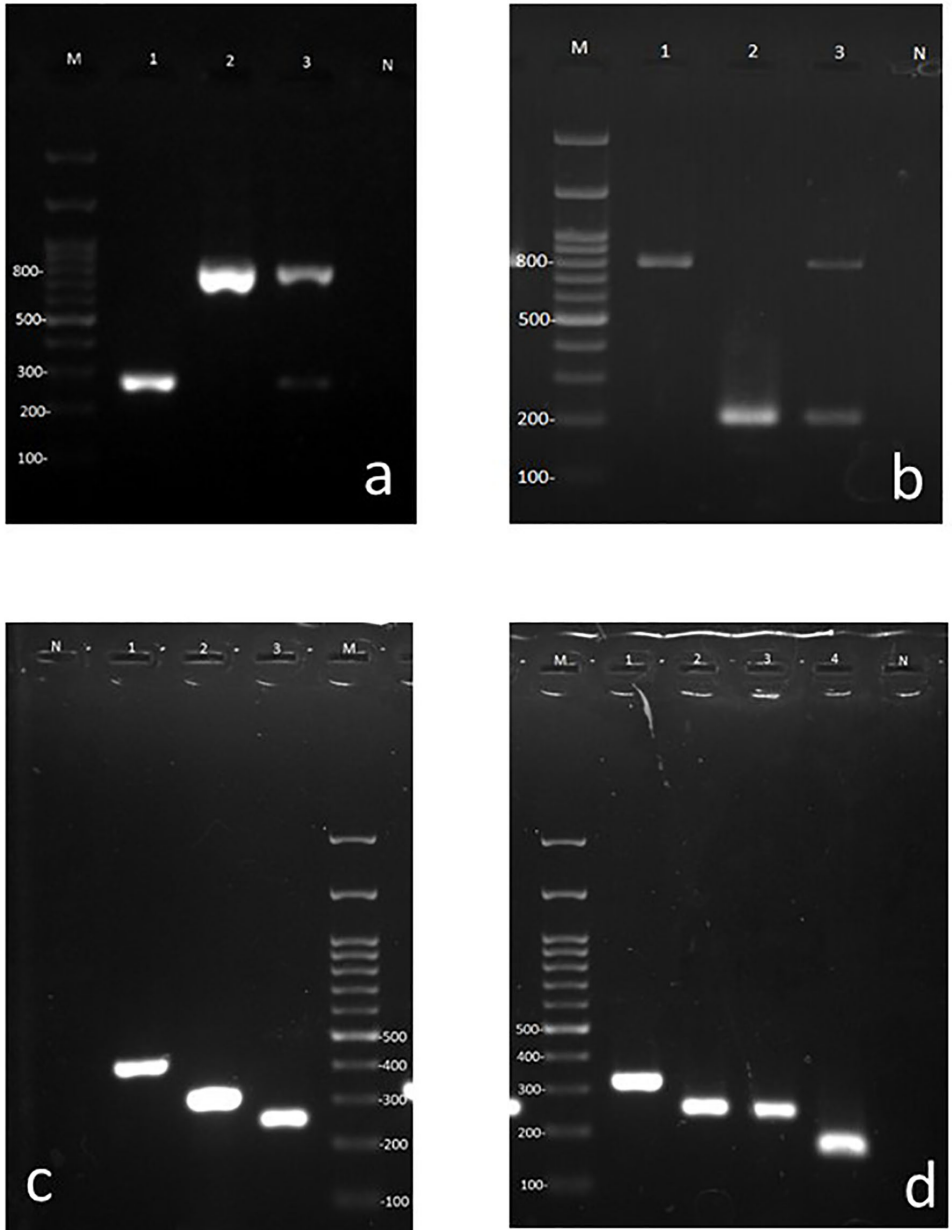
Agarose gels containing representative amplicons. **(A)** First step multiplex PCR using universal bacterial and fungal primers. Lane M 100 bp ladder, Lane 1: Bacterial (265–267 bp band), Lane 2: Fungal (783–800 bp band), Lane 3: Fungal and bacterial co-infection (dual bands of 265–267 and 783–800 bps), Lane N: negative control for PCR. **(B)** *Pseudomonas* and *Staphylococcus* specific primer pairs (bacterial duplex PCR), Lane M: 100 bp ladder, Lane 1: *Staphylococcus*, Lanes 2: *Pseudomonas*, 3: mixed of *Pseudomonas* and *Staphylococcus* (dual bands of 198 and 756 bp, respectively), Lane N: negative control for PCR. **(C)** *Candida* species specific multiplex primers. Lane N: negative control for PCR, Lane1: *C. albicans*, Lane 2: *C*. *parapsilosis*, Lane 3: *C*. *auris*, Lane M: 100 bp ladder. **(D)** *Aspergillus* species specific multiplex primer. Lane M: 100 bp ladder, Lane 1: *A*. *terreus*, Lane 2: *A*. *tubingensis*, Lane 3: *A*. *niger*, Lane 4: *A*. *flavi*, Lane N: negative control for PCR.

A total of 120 frozen specimens from patients suspected of microbial OE which were previously shown to contain pure fungal and bacterial elements in 21 and 12 specimens, respectively, along with 76 specimens which were demonstrated to be simultaneously infected with bacterial (n = 88) and fungal (n = 97) elements using direct examination or Giemsa staining were subjected to DNA extraction and PCR in a blinded way. Out of 120 specimens included in this study, the overall multiplex PCR based detection rate of bacterial (n = 88; 73.3%) and fungal (n = 97; 81%) OE documented is 100%, of which 21 and 12 specimens yielded single amplicon band of 783–800 and 265–267 bp corresponding to fungal and bacterial targets, respectively (**Figure 2A**). Accordingly, the co-infection of bacterial and fungal pathogens (two amplicon bands were simultaneously yielded) by the first step of multiplex PCR was demonstrated in 76 specimens (63.3%) (**Figure 2A**). Therefore, the detection rate (sensitivity) of pan-fungal/pan-bacterial multiplex PCR showed complete consistency with the previously documented results of the direct examination. Considering the results of the first step multiplex PCR, an amplicon size of 265–267 bp implying bacterial agents was revealed in 88 specimens. Therefore, the specimens whose etiological agents were diagnosed to be bacterial OE were subjected to a second stepwise bacterial multiplex PCR identifying *Staphylococcus* and *Pseudomonas* genera. Out of 88 specimens with positive pan-bacterial PCR, the identification of agents was successfully achieved for 83 specimens. Accordingly, analysis of the amplicon size showed that *Staphylococcus* and *Pseudomonas* were the causative agents in 36 and 17 specimens, respectively. In addition, for 30 specimens, two amplification bands were simultaneously observed implying the co-infection of *Staphylococcus* and *Pseudomonas* (**Figure 2B**). The identification of the rest of the specimens (~6%) could not be provided. Therefore, *Staphylococcus* was evidenced as the most predominantly identified genus causing bacterial OE accounting for 75% of the cases with bacterial OE.

### PCR *Versus* Culture for Detection/Identification of Bacterial and Fungal OE

Regarding the previously documented results of direct examination, which showed fungal elements in 97 specimens, the positivity rate of the first step of the PCR detecting fungal agents causing OE showed to be higher than that in the results previously obtained by culture (positive culture in 81 specimens). All 81 (83.5%) samples with culture positive for fungi were pan-fungal PCR positive and showed complete agreement. However, specimens that demonstrated positive bacterial elements in direct examinations in our previous study had not been investigated by culture media; therefore, the related results of culture were not available for comparison with the positivity rate of pan-bacterial PCR. It should be noted that 16 specimens with negative fungal culture were also successfully amplified in pan fungal PCR. Direct examinations of 12 specimens showed the clusters of single and broad base budding yeast cells, implying *Malassezia* species; moreover, four cases revealed septate hyphal elements that became *Aspergillus* positive in PCR.

### Basic Molecular Versus Multiplex PCR for Fungal Identification

The identification of fungal agents which were investigated through the second stepwise multiplex PCR was documented to be 92.4% (47 *Aspergillus* and 26 *Candida* species). Moreover, it was consistent with the identification results obtained previously by PCR-RFLP, ITS, or *β*-tubulin gene sequencing of 79 isolates (53 *Aspergillus* and 26 *Candida*) recovered from the culture. Nonetheless, the *Aspergillus*-related multiplex PCR of six out of 53 specimens with culture positive for *Aspergillus* species did not yield an amplicon band. On the other hand, our designed *Aspergillus*-related multiplex PCR was able to identify four culture-negative specimens with direct examination indicating fungal hyphae. Considering the amplicon size of the first multiplex PCR covering the detection of fungal and bacterial OE, 97 specimens showed a 783–800 bp size band corresponding to fungal pathogens. Subsequently, the specimens whose etiological agents were diagnosed as fungi were subjected to *Candida-* and *Aspergillus-*related multiplex PCR, separately. In total, 67 and 51 specimens contained *Candida* and *Aspergillus* species, respectively (**Figures 2C, D**), among which 30 specimens showed dual amplicon bands and were simultaneously positive with both *Candida* and *Aspergillus* related multiplex PCR. However, according to the previously documented results of ear aspiration cultures, 27 and 53 isolates of *Candida* and *Aspergillus* species were recovered and molecularly identified, respectively (Aboutalebian et al., 2019). According to the results of the second fungal multiplex PCR, *C. albicans*, *C. parapsilosis*, and *A.* Section *Nigri* were recorded as the most predominantly identified species in descending frequency among fungal agents infecting the ear canal. However, this frequency should be interpreted with caution since *Candida* species can be a member of normal flora or colonizer of the external ear canal and the population of *Candida* may be increased during antibacterial therapy.

## Discussion

Delayed or blind treatment of infectious EO due to misdiagnosis or delayed detection may cause myringitis, auricular cellulitis, and otitis media (Adegbiji et al., 2017). Therefore, timely and definitive diagnosis through fast, reliable, and sensitive methods enables the tailoring of appropriate treatment, thereby abolishing avoidable complications (Musso and Crews, 2016; Adegbiji et al., 2017). Given the great number of mixed fungal and bacterial ear infections, the current study investigated the diagnostic value of pan-fungal/pan-bacterial multiplex PCR in order to differentially detect fungal and bacterial OE in the ear specimens aspirated from patients previously proven to carry fungal and bacterial ear infections. One by one pathogen targeting is time-consuming, and contamination-susceptible PCR set-ups make the approaches infeasible for routine laboratory (Matar et al., 1998). One of the great advantages of the multiplex PCR is that this technique provides the simultaneous detection of several species and mixed infections with smaller amounts of reagents and samples (Hendolin et al., 2000). Due to the substantial importance of accurate identification of the causal agent for targeted therapy (Blomgren and Pitkäranta, 2005; Ayub et al., 2015; Adegbiji et al., 2017), *Staphylococcus* and *Pseudomonas* specific primers, as well as *Candida* and *Aspergillus* species-specific primers, the most frequently encountered agents implicated in OE infection (Aboutalebian et al., 2019) were assessed in a stepwise multiplex PCR manner in this study. No similar studies have investigated so far to molecularly differentiate the fungal and bacterial agents affecting the ear canal. To the best of our knowledge, the current study is the first report aimed to discriminate bacterial and fungal etiology of ear infections through a stepwise multiplex PCR manner. The distinction of the etiological agents affecting the ear canal is a crucial issue to ensure the accurate choice of drug warranting the successful therapy considering the different management and variations of the *in vitro* antimicrobial susceptibility patterns among different fungal and bacterial genera (even different species belonging to the same genus), emergence of infections caused by *C. auris*, fewer susceptible species to routinely used antifungal agents, and differential diagnosis (Adegbiji et al., 2017; Abastabar et al., 2019; Aboutalebian et al., 2020). The efficiency of the multiplex PCR assay for simultaneous detection of fungi and bacteria was addressed by comparing its results with those of direct examination and culture. Since a great number of our samples showed bacterial and fungal co-infection *via* universal primers, combined antifungal and antibacterial approach therapy may be necessary in these patients. The overall multiplex PCR-based detection rate of bacterial (n = 88; 73.3%) and fungal (n = 97; 81%) OE was documented to be 100%. Moreover, the detection rate (sensitivity) of pan-fungal/pan-bacterial multiplex PCR showed complete consistency with the previously documented results of direct examination and Giemsa staining (Aboutalebian et al., 2019). Furthermore, the positivity rate of the first step of the PCR detecting fungal agents showed to be higher than that in the results previously obtained by culture (100 *vs.* 83.5%). Culture-based therapy is essential for the successful resolution of agents causing the infection in most cases particularly those who have an inadequate response to treatment (Gruber et al., 2015; Musso and Crews, 2016). The PCR assay was demonstrated to be more sensitive, more rapid, and less cumbersome than culture (74 *vs.* 21%) (Matar et al., 1998). Nevertheless, in case of slow-growing or fastidious bacteria or fungi, pretreatment antimicrobial, low bacterial or fungal count, unavailability of fungi and bacteria due to biofilm formation, or/and polymicrobial colonization of the ear canal, the results of culture can be misleading and less sensitive (Post, 2001; Jalali et al., 2008; Hasibi et al., 2017). In our previous study, bacterial media culture was not used to recover bacterial agents; however, 83.5% of 97 specimens with positive direct examination yielded fungal growth in fungal media culture (Aboutalebian et al., 2019). Culture is necessary to recover the infecting strain, identify the species level, and assess its antifungal susceptibility pattern (Rios-Yuil, 2016; Aboutalebian et al., 2019). However, obligate lipid-dependent *Malassezia* species cannot easily be recovered by the routinely used fungal culture (Konaté et al., 2017). In our previous study (Aboutalebian et al., 2019), 12 specimens had clusters of single and budding yeast-like cells mixed with short and narrow hyphal fragment in direct examination suggestive of *Malassezia* species; moreover, their culture remained negative but pan-fungal PCR provided positive results for them. Therefore, the necessity for culture-based molecular identification will be eliminated with a direct PCR on the clinical specimen (Rios-Yuil, 2016). In our study, about 16.5% discordance was noted between the pan-fungal PCR and the fungal culture data, which confirms greater sensitivity of PCR compared to culture-based methods. These results are supported by the finding of other studies, as it was demonstrated that 77.3% of the ear aspiration specimens were PCR positive for one or more organisms in tests with viral and bacterial primers (Post et al., 1998). In another study, a two-step PCR-based assay using pan-bacterial and *Haemophilus*, as well as *Streptococcus* and *Moraxella catarrhalis* specific primers was performed to detect the bacterial etiologies of otitis media with effusion in Lebanese children. According to the results, 53% discordance was revealed between PCR results and those of culture (Matar et al., 1998). Similarly, although only 20–30% of ear effusions yielded positive results through culture, up to 75% of the specimens had positive PCR for pathogenic bacteria (Hendolin et al., 2000; Gok et al., 2001). In another study, the PCR performed for the detection of fungal DNA in the middle ear effusion of patients suffering from otitis media showed to be more sensitive (29 *vs.* 8%) than fungal culture (Jalali et al., 2008). Our results imply that the multiplex PCR is more efficient than culture in detecting the DNA of bacterial and fungal agents in EA samples. However, since both are viable and fossil of the organism’s genetic material can be amplified by PCR, residual DNA from a previous episode of OE infection is probably detected as well (Post et al., 1996). The experimental model has shown that the bacterial DNA in the ear aspiration samples disintegrates within two days following bacterial death (Matar et al., 1998). Noteworthy, in all positive pan-bacterial PCR (n = 88), bacterial elements were evidenced with Giemsa staining. Therefore, it is probable that the bacterial DNA detected by the universal primers in our EA samples might have originated from viable bacteria implicated in disease and not residual DNA persisting from a former episode of OE infection. In the same line, our results have shown the presence of fungal and bacterial DNA in 81 and 73.3% of the samples (n = 120), respectively. The rate of fungal OE was higher than that of bacterial OE, and it is also above the rate reported earlier (Lee et al., 2008). The high rate of fungal OE can be attributed to the fact that the cases suspected to carry otomycosis were recruited in our previous study (Aboutalebian et al., 2019). However, some of the specimens showed to be infected with either bacterial agent as mono-caused OE infection or mixed fungal and bacterial infection. Considering the results of species identification through our genus and species-specific primers, our finding showed that about 95% of bacterial OE is caused by *Staphylococcus* and *Pseudomonas*. The identification of *Staphylococcus* and *Pseudomonas* genera was only investigated in this study since previous studies have reported that 92% of patients with bacterial EO were implicated by *Staphylococcus* and *Pseudomonas* genera (Clark et al., 1997). The isolation rate of non-*Staphylococcus*/*Pseudomonas* genera is documented in only 5–20% of bacterial OE (Clark et al., 1997; Musso and Crews, 2016). The high rate of PCR positive by *Staphylococcus* specific primer (75%) could be attributed to the bacterial flora residing in the ear canal (Musso and Crews, 2016); however, since in all experiments, control panels, including ear samples, obtained from healthy individuals (served as negative control) were tested into the assay system, the likelihood of false positivity is disregarded. Moreover, our subjects were clinically relevant cases of ear infection (Aboutalebian et al., 2019). Regarding the prognosis of *Aspergillus* and *Pseudomonas* OE that are more associated with the spread and involve the temporal bone, skull base, and multiple cranial nerves, especially in susceptible hosts, and cause a disorder termed as necrotizing (malignant) OE (Musso and Crews, 2016), the importance of identification of the species causing the disease is doubled. *Aspergillus* and *Candida* species particularly those belonging to the complexes *A.* Section *Nigri*, *A.* section *flavi*, and *C. parapsilosis*, are the most prevailing causes of otomycosis (Musso and Crews, 2016; Aboutalebian et al., 2019). Additional fungi less commonly reported to be implicated in otomycosis include *Cladosporium, Penicillium, Alternaria, Cryptococcus*, *Talaromyces* (Aboutalebian et al., 2020), and *C. auris* (Abastabar et al., 2019). According to the results of the second fungal multiplex PCR, *C. albicans*, *C. parapsilosis*, and *A.* Section *Nigri* were recorded as the most predominantly identified species in descending frequency. However, culture-based molecular identification showed that species under *A.* Section *Nigri* and then *C. parapsilosis* were the most frequent causal agents (Aboutalebian et al., 2019). Considering the lack of significant difference in the antifungal susceptibility patterns of different species categorized in different *Aspergillus* sections (Szigeti et al., 2012), the identification of the related species up to section level was regarded in this study. The high rate of positive *Candida*-related multiplex PCR and specimens simultaneously showed dual amplicon bands of *Candida* and *Aspergillus* species possibly due to *Candida* overgrowth and colonization in unhealthy and humidity accumulated ear (Musso and Crews, 2016; Aboutalebian et al., 2019) or dual infection. In cases of co-infection, multiplex PCR assays targeting different genera or species-specific regions have been demonstrated to be more sensitive than culture for accurate discrimination and identification of the fungi (Zaman et al., 2017). Given the increasing reports of otomycosis due to *C. auris* (Abastabar et al., 2019; Jung et al., 2020), as well as the emerging multidrug-resistant yeast that was erroneously identified as *C. parapsilosis*, C. *haemulonii, C. famata*, and *Rhodotorula glutinis* through conventional and biochemical assays, specific primers were designed capturing *C. auris* in the heart of *Candida* related multiplex PCR. This is one of the strengths of the present study. Interestingly, two ear samples were positive for *C. auris* in the species-specific multiplex PCR of *Candida*; however, *C. auris* colonies were not isolated in this study, and they require more investigations before reporting. The sensitivity of identification of *Aspergillus* species directly from fresh specimens was lower than culture-based molecular identification, indicating the possibility that in clinical specimens, DNA is damaged during tissue processing and DNA extraction. Molecular diagnosis and species identification by universal and species-specific primers had a lower turnaround time (~10 h), compared to culture, sequencing, and histopathology. This finding is important for early diagnosis followed by targeted therapy (Blomgren and Pitkäranta, 2005; Thorne and Wetmore, 2009; Ayub et al., 2015; Musso and Crews, 2016).

## Conclusion

This study revealed simultaneous detection and partial identification of fungal, bacterial, and mixed OE infections from clinical ear specimens with smaller amounts of reagents and samples using pan-fungal/pan-bacterial multiplex PCR. The sole dependence on conventional and phenotypical assays could result in misdiagnosis and erroneous identification and oblivion of emerging yeast species, such as *C. auris*. The necessity for culture based-molecular identification will be eliminated through stepwise molecular detection/identification directly from clinical samples; accordingly, misidentification, turnaround time, and expenses will be reduced in this regard. The stepwise multiplex PCR assay is recommended as a better detector of agents implicated in fungal and bacterial OE.

## Data Availability Statement

The raw data supporting the conclusions of this article will be made available by the authors, without undue reservation.

## Ethics Statement

This study was reviewed and approved by the national institute for medical research development (NIMAD) by the grant number: 982931, and the ethics code: IR.NIMAD.REC.1398.063. The patients provided their written informed consent to participate in the study.

## Author Contributions

SA and HM have designed the study. SA did the experiments. AO contributed in patient selection, clinical diagnosis, and treatment of the subjects. KA, HF, and HM participated in draft preparation and revision. All authors assisted in paper edition and revision. MN, JC, and HM have provided the isolates, participated in carrying out the experiments, and assisted in paper revision. SA participated in performing multiplex PCR. All authors contributed to the article and approved the submitted version.

## Conflict of Interest

The authors declare that the research was conducted in the absence of any commercial or financial relationships that could be construed as a potential conflict of interest.
